# Microarray profiling analysis and validation of novel long noncoding RNAs and mRNAs as potential biomarkers and their functions in atherosclerosis

**DOI:** 10.1152/physiolgenomics.00077.2019

**Published:** 2019-11-04

**Authors:** Huan-Lan Bai, Zhi-Feng Lu, Jing-Jing Zhao, Xin Ma, Xue-Heng Li, Hui Xu, Shao-Guo Wu, Chun-Min Kang, Jing-Bo Lu, Yuan-Jun Xu, Lei Xiao, Qian Wu, Shu Ye, Qian Wang, Lei Zheng, Yan-Wei Hu

**Affiliations:** ^1^Laboratory Medicine Center, Nanfang Hospital, Southern Medical University, Guangzhou, Guangdong, China; ^2^Department of Anesthesiology, Nanfang Hospital, Southern Medical University, Guangzhou, Guangdong, China; ^3^The Qingyuan Traditional Chinese Medical Hospital of Guangdong Province, Qingyuan, Guangdong, China; ^4^Department of Clinical Laboratory, Guangzhou Twelfth People’s Hospital, Guangzhou, Guangdong, China; ^5^Department of Vascular Surgery, Nanfang Hospital, Southern Medical University, Guangzhou, Guangdong, China; ^6^Department of Cardiovascular Sciences, University of Leicester, Leicester, United Kingdom; ^7^Shantou University Medical College, Shantou, Guangdong, China; ^8^Laboratory Medicine Center, Guangzhou Women and Children's Medical Center, Guangzhou, Guangdong, China

**Keywords:** atherosclerosis, biomarker, lncRNA, microarray analysis

## Abstract

Long noncoding (lnc)RNAs have been implicated in the development and progression of atherosclerosis. However, the expression and mechanism of action of lncRNAs in atherosclerosis are still unclear. We implemented microarray analysis in human advanced atherosclerotic plaques and normal arterial intimae to detect the lncRNA and mRNA expression profile. Gene Ontology functional enrichment and pathway analyses were applied to explore the potential functions and pathways involved in the pathogenesis of atherosclerosis. A total of 236 lncRNAs and 488 mRNAs were selected for further Ingenuity Pathway Analysis. Moreover, quantitative RT-PCR tests of most selected lncRNAs and mRNAs with high fold changes were consistent with the microarray data. We also performed ELISA to investigate the corresponding proteins levels of selected genes and showed that serum levels of SPP1, CD36, ATP6V0D2, CHI3L1, MYH11, and BDNF were differentially expressed in patients with coronary heart disease compared with healthy subjects. These proteins correlated with some biochemical parameters used in the diagnosis of cardiovascular diseases. Furthermore, receiver operating characteristic analysis showed a favorable diagnostic performance. The microarray profiling analysis and validation of differentially-expressed lncRNAs and mRNAs in atherosclerosis not only provide new insights into the pathogenesis of this disease but may also reveal new biomarkers for its diagnosis and treatment.

## INTRODUCTION

Atherosclerosis is the common pathological basis of coronary heart disease and many other cardiovascular diseases (3, 17). The pathogenesis of atherosclerosis is complex, involving lipid deposition, vascular smooth muscle cell proliferation and apoptosis, endothelial cell dysfunction, and macrophage foam cell formation (7, 9). A large number of molecules such as transcription factors, cytokines, and kinases participate in this progress.

Long noncoding RNAs (lncRNAs) are noncoding RNAs more than 200 nucleotides in length located in the cell nucleus or cytoplasm (19, 30). With the exploration of lncRNAs, several hypotheses that lncRNAs are associated with mRNA expression have been proposed (27). lncRNAs can interfere with nearby promoters by cis- or trans-targeting, causing transcriptional interference (6, 16, 19). As an enhancer, lncRNA stabilizes chromatin loops, leading to a distal enhancer to interact with a promoter. In posttranscriptional regulation, lncRNAs combine with mRNA through microRNAs (miRNAs) to synthesize lncRNA-miRNA-mRNA axis, affecting mRNA stability or translation. Additionally, lncRNAs act as miRNA precursors or miRNA sponges to regulate corresponding miRNA, affecting in posttranscriptional regulation of gene expression (15, 24). Moreover, lncRNAs are also involved in many biological processes such as chromatin modification, transcription, splicing, translation, degradation, and transport and have effects on growth, metabolism, and senescence through cell proliferation, differentiation, and apoptosis (8, 31, 33, 34, 41). Accumulating studies indicate that lncRNAs are involved in the process of injury and repair of endothelial cells, migration and proliferation of smooth muscle cells, lipid loading and inflammatory response in macrophages, and lipid deposition and the formation of plaques, all of which affect the progression of atherosclerosis and other cardiovascular diseases (4, 21, 23). For example, the lncRNA ANRIL (antisense noncoding RNA in the INK4 locus) is associated with coronary atherosclerosis, carotid atherosclerosis, and peripheral artery disease (1, 11, 26). The lncRNA MALAT1 (metastasis-associated lung adenocarcinoma transcript 1), on the other hand, has reduced expression in patients with coronary artery disease (1, 10). Using microarray analysis, we recently found that the lncRNA RP5-833A20.1/has-miR-382-5p/nuclear factor IA pathway is essential for the regulation of cholesterol homeostasis and inflammatory responses in THP-1 macrophages (14). Furthermore, we found that lncRNA NEXN-AS1 (nexilin F-actin binding protein antisense RNA 1) inhibited TLR4 oligomerization and NF-κB activity, suppressed monocyte adhesion to endothelial cells, and prevented against atherosclerosis (13). However, studies of lncRNA in atherosclerosis are in their infancy, and our understanding of the role of lncRNA in this disease remains limited.

To systematically study the role of lncRNAs in atherosclerosis, we examined lncRNA and mRNA expression profiles in samples of atherosclerotic plaque and normal arterial intima. This study was designed to identify lncRNAs and mRNAs implicated in the pathogenesis of atherosclerosis and to establish candidate biomarkers for prevention and treatment of atherosclerosis.

## MATERIALS AND METHODS

### 

#### Patients and samples.

For microarray analysis, six tissue samples were collected from patients at Nanfang Hospital, Guangzhou, China. According to the 1990 classifications of the Committee on Vascular Lesions of the Council on Atherosclerosis, American Heart Association, advanced abdominal aortic plaques or common carotid plaques were taken from patients diagnosed with grade V or VI atherosclerosis. As a normal arterial intima control, the abdominal aortas of three patients without atherosclerosis were obtained. One of the normal arterial intimae and advanced plaque tissue were collected from the same individual. Before the first surgery, no patient had received previous atherosclerotic treatment. Samples were obtained and frozen in liquid nitrogen. To confirm and validate the expression of differentially expressed lncRNAs and mRNAs in clinical samples, we also obtained 10 normal intima, and 15 plaque tissues from patients at Nanfang Hospital matched in age and sex. Between October 2015 and July 2016, serum samples were collected from patients with chronic coronary heart disease (CCHD; *n* = 108), acute coronary syndrome (ACS; *n* = 93), and heart failure (HF; *n* = 75), as well as from healthy controls (*n* = 103). Diagnosis of these cardiovascular diseases was made according to the clinical guidelines standardized by the American College of Cardiology/American Heart Association (22, 39a). Patients diagnosed with any form of cancer were excluded from the study group. Serum was collected before treatment with any medications and stored at −80°C. Basic clinical test results were also collected including serum total cholesterol (TC), triglyceride (TG), high density lipoprotein (HDL), low density lipoprotein (LDL), apolipoprotein A-I (ApoA-1), ApoB, lipoprotein a (LPa), homocysteine (HCY), myoglobin (Myo), cardiac troponin I (cTnI), creatine kinase (CK), CK-MBmass (CK-MBm), CK-MB, C-reactive protein (CRP), lactate dehydrogenase (LDH), and aspartate transaminase (AST). All samples were collected with informed written consent from patients, and ethical consent was granted from the Committees for Ethical Review of Research involving Human Subjects of Southern Medical University, Guangzhou, China([2013]111).

#### Microarray analysis.

In accordance with the manufacturer’s instructions, total RNA was extracted from frozen tissue with TRIzol reagent (Invitrogen, Carlsbad, CA). RNA quality was assessed by NanoDrop ND-1000, while RNA integrity was evaluated with standard denaturing agarose gel electrophoresis. Microarray analysis was performed with the Agilent Array platform (Agilent Technologies). Under the manufacturer’s standard protocols with minor modifications, sample preparation and microarray hybridization were performed. In short, mRNA was purified from total RNA after removal of rRNA (mRNA-ONLY Eukaryotic mRNA Isolation Kit, Epicenter). Thereafter, employing a random primer method, we amplified all samples and transcribed them into fluorescent cRNA along the whole length of the transcript without 3′-bias. The labeled cRNAs were hybridized onto the Human lncRNA Array v3.0 (8 × 60K, Arraystar). After washing the slides, we scanned the arrays with the Agilent Scanner G2505C. Agilent Feature Extraction software (version 11.0.1.1) was utilized to analyze acquired array images, and the GeneSpring GX v11.5.1 software package (Agilent) was applied to quantile normalization and subsequent data processing. LncRNAs and mRNAs from at least three out of six samples flagged as present or marginal (“All Targets Value”) were chosen for further data analysis after quantile normalization of the raw data. Volcano plot filtering was applied to identify the differentially expressed lncRNAs and mRNAs statistically different between the two groups. The roles these differentially expressed mRNAs play in terms of biological pathways or gene ontology (GO) terms were determined through pathway analysis and GO analysis. Finally, the distinguishable lncRNA and mRNA expression patterns among samples were showed by hierarchical clustering. All of the microarray data were upload to the National Center for Biotechnology Information Gene Expression Omnibus (GEO) with a GEO accession number (GSE97210).

#### Ingenuity Pathway Analysis.

Biological function enrichment, canonical pathway analysis, and upstream regulator analysis were identified applying the Ingenuity Pathway Analysis database (IPA, Ingenuity Systems Inc., Redwood City, CA).

#### Network analysis.

GeneGo MetaCore data mining software was applied to construct gene networks. Enrichment analysis included mapping gene IDs of the data set onto gene IDs in entities of built-in functional ontologies represented in MetaCore by pathway maps and networks. IPA linked specific genes to a database of gene functions gleaned from the biomedical research literature and was used to generate additional network associations. More information about the two kinds of software was acquired from http://www.geneontology.org/ and https://www.ingenuity.com.

#### RNA isolation and real-time quantitative PCR analysis.

Total RNA from tissues was extracted with TRIzol reagent (TaKaRa Bio, Inc., Shiga, Japan) based on the manufacturer’s protocol. Real-time PCR was performed on a real-time PCR ABI 7500 Fast System (Applied Biosystems, Foster City, CA). LncRNA levels were assessed by SYBR Green qPCR super Mix (Invitrogen). The expression of U6 RNA was used as an internal control. And the ABI 7500 Fast Real-Time PCR system with SYBR Green detection chemistry (TaKaRa) was performed to evaluate mRNA levels. Glyceraldehyde 3-phosphate dehydrogenase expression was used as an endogenous control. The quantitative measurement was determined by the ^ΔΔ^Ct method. Each sample was measured in triplicate and the mean value was considered for comparative analysis. The primers are listed in Supplementary Table S1 (all supplementary tables are available at https://figshare.com/articles/Supplemental_Tables_docx/9600689).

#### Measurement of serum proteins selected from microarray analysis.

The expression levels of selected proteins were measured by enzyme-linked immunosorbent assay (ELISA) based on the manufacturer’s instruction. Commercial ELISA kits were purchased from different companies listed in Supplementary Table S2.

#### Statistical analysis.

Data analyses were performed using Statistical Package for the Social Sciences (version13.0) software (SPSS, Inc, Chicago, IL). Data are presented as means ± SD or medians (interquartile range) unless otherwise indicated. Results were analyzed by one-way analysis of variance followed by the Bonferroni test, or by an unpaired Student’s *t* test when continuous variables were normally distributed, then multiple testing correction was adopted by Benjamini and Hochberg false discovery rate. When the data were not normally distributed, the Mann-Whitney *U* test was utilized to analyze continuous variables. The correlations between selected proteins levels and biochemical parameters were determined by Pearson or Spearman correlation analysis. After adjusting for sex, age, and known cardiovascular risk factors to estimate the magnitude of associations between the selected proteins and cardiovascular diseases, we employed logistic regressions to calculate the odds ratio (OR) and 95% confidence interval (CI). The receiver operating characteristic (ROC) curve was generated to determine the diagnostic performance of selected proteins. A two-tailed probability *P* value <0.05 was considered statistically significant.

## RESULTS

### 

#### Overview of lncRNA and mRNA expression profiles.

**Fig. 1.**
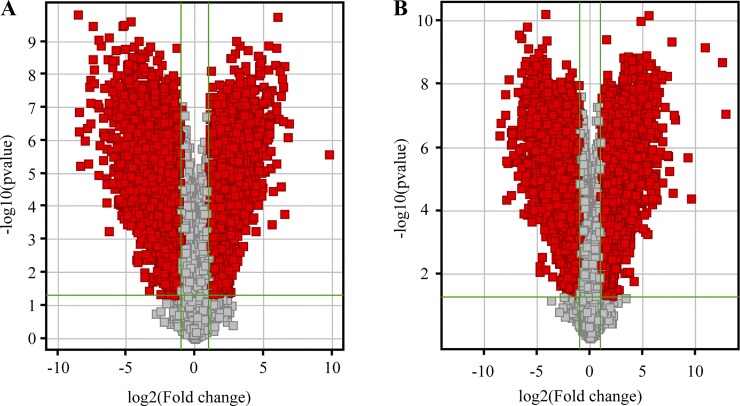
Profiles of lncRNA and mRNA microarray data. Volcano plots of lncRNA expression profiles (*A*) and mRNA expression profiles (*B*) were used for visualizing differential expression between two different conditions. Each red point in the plot represents differentially expressed RNA with statistical significance. The vertical lines represent 2.0-fold up- and downregulation and the horizontal lines indicate a *P* value of 0.05.

In this research, we have characterized the lncRNA and mRNA profiles in three advanced atherosclerosis samples and in three normal intima tissues. A total of 30,586 lncRNAs and 26,109 coding transcripts were detected by microarray analysis. As shown in Fig. 1, volcano plot filtering found that 5,461 lncRNAs were upregulated and 6,115 lncRNAs were downregulated in advanced atherosclerotic plaques compared with normal arterial intima (fold change >2 and *P* < 0.05; Fig. 1*A*). On the other hand, 4,230 mRNAs were upregulated and 4,128 mRNAs were downregulated (Fig. 1*B*). Scatter plots were used to visualize the expression variation between the two groups and showed a >2.0-fold change in abundant lncRNAs and mRNAs (Supplementary Fig. S1, A and B; all supplementary figures are available at https://figshare.com/articles/Supplementary_Figures/9601130). Hierarchical clustering exhibits the relationships among lncRNAs as well as mRNA expression patterns present in the samples (Supplementary Fig. S1, C and D). Based on the magnitude of change, we listed the top 10 upregulated and downregulated lncRNAs and mRNAs in Table 1 and Table 2.

**Table 1.** Top 10 upregulated and downregulated lncRNAs in advanced atherosclerotic plaques compared with normal arterial intimae

Seqname	Gene Symbol	Regulation	*P* Value	Absolute Fold Change	RNA Length	Chromosome	Strand	Relationship
uc001gzl.3	BC034684	up	2.59323E-06	885.5702623	460	chr1	−	exon sense-overlapping
ENST00000445003	RP11-290F20.3	up	3.00834E-07	116.3594845	539	chr20	+	intergenic
ENST00000443523	RP11-556E13.1	up	0.000172926	93.78290522	369	chr10	−	intergenic
ENST00000569037	RP11-212I21.2	up	5.44075E-09	89.72674683	553	chr16	−	intronic antisense
ENST00000437416	RP11-100E13.1	up	2.31625E-08	89.05720113	403	chr1	−	bidirectional
ENST00000540226	RP11-598F7.3	up	2.66734E-07	87.3681841	586	chr12	−	intronic antisense
ENST00000557062	RP11-816J8.1	up	1.34352E-08	82.92993176	522	chr14	−	intergenic
uc001zvk.3	HMGN2P46	up	2.387E-05	77.29568107	942	chr15	+	natural antisense
ENST00000432711	AC064834.3	up	4.38278E-08	77.22978411	555	chr2	−	intergenic
ENST00000510230	CTB-138E5.1	up	7.75597E-09	74.97468757	504	chr5	+	intronic antisense
uc011mfp.1	AK127309	down	1.44066E-10	360.6950965	3067	chrUn_gl000219	−	intergenic
ENST00000453118	RP1-163G9.1	down	5.21846E-07	347.0138096	1697	chr1	−	intergenic
ENST00000512263	RP11-161D15.1	down	1.38021E-07	344.6481593	499	chr4	+	intergenic
ENST00000562459	CTD-3064M3.3	down	5.75701E-06	321.3428213	1963	chr8	−	intergenic
NR_024344	LOC283174	down	1.02876E-06	269.4272507	5306	chr11	−	intergenic
ENST00000502941	RP11-471J12.1	down	4.04957E-07	238.7432554	358	chr4	+	bidirectional
ENST00000564832	RP11-531A24.3	down	5.11986E-06	222.4989781	3296	chr8	+	intergenic
NR_027180	MIR143HG	down	2.24646E-08	205.9827099	9070	chr5	+	intergenic
uc003lqr.3	BX640700	down	6.93259E-08	197.5611096	5117	chr5	+	intergenic
ENST00000563424	RP11-401P9.4	down	2.93404E-07	195.7111309	3441	chr16	+	intergenic

**Table 2.** Top 10 upregulated and downregulated mRNAs in advanced atherosclerotic plaques compared with normal arterial intimae

Seqname	Gene Symbol	Regulation	*P* Value	Absolute Fold Change	Regulation	RNA Length	Chromosome	Strand
NM_001040060	SPP1	up	8.48553E-08	7316.686689	up	1560	chr4	+
NM_002423	MMP7	up	4.05031E-05	762.442449	up	1147	chr11	−
NM_018643	TREM1	up	1.98707E-06	606.5559703	up	1633	chr6	−
ENST00000368738	S100A9	up	1.29326E-07	255.8468333	up	577	chr1	+
NM_001001548	CD36	up	8.96768E-08	250.779817	up	4727	chr7	+
NM_138461	TM4SF19	up	2.73667E-05	223.2345985	up	1077	chr3	−
NM_001444	FABP5	up	4.33327E-10	210.793027	up	751	chr8	+
NM_152565	ATP6V0D2	up	5.68548E-06	177.0426041	up	2370	chr8	+
NM_003467	CXCR4	up	1.88792E-06	171.8156451	up	1691	chr2	−
NM_001276	CHI3L1	up	5.398E-09	161.2576396	up	1867	chr1	−
NM_001146312	MYOCD	down	4.15082E-07	388.0686464	down	6950	chr17	+
NM_182826	SCARA3	down	1.66378E-07	347.4284531	down	1711	chr8	+
NM_203371	FIBIN	down	9.53543E-08	270.8993334	down	3024	chr11	+
NM_001017398	TRIM36	down	2.14195E-08	269.141173	down	906	chr5	−
NM_001608	ACADL	down	2.62191E-06	236.8372231	down	2565	chr2	−
ENST00000377694	IGFBPL1	down	3.15538E-06	224.5009801	down	1093	chr9	−
NM_002474	MYH11	down	6.73347E-09	213.8021672	down	6882	chr16	−
NM_024686	TTLL7	down	3.30751E-07	187.8536755	down	3648	chr1	−
NM_170734	BDNF	down	3.24577E-05	183.3155043	down	3976	chr11	−
NM_053027	MYLK	down	8.07202E-07	183.1613974	down	7699	chr3	−

#### GO and Kyoto Encyclopedia of Genes and Genomes pathway analysis of differentially-expressed mRNAs.

**Fig. 2.**
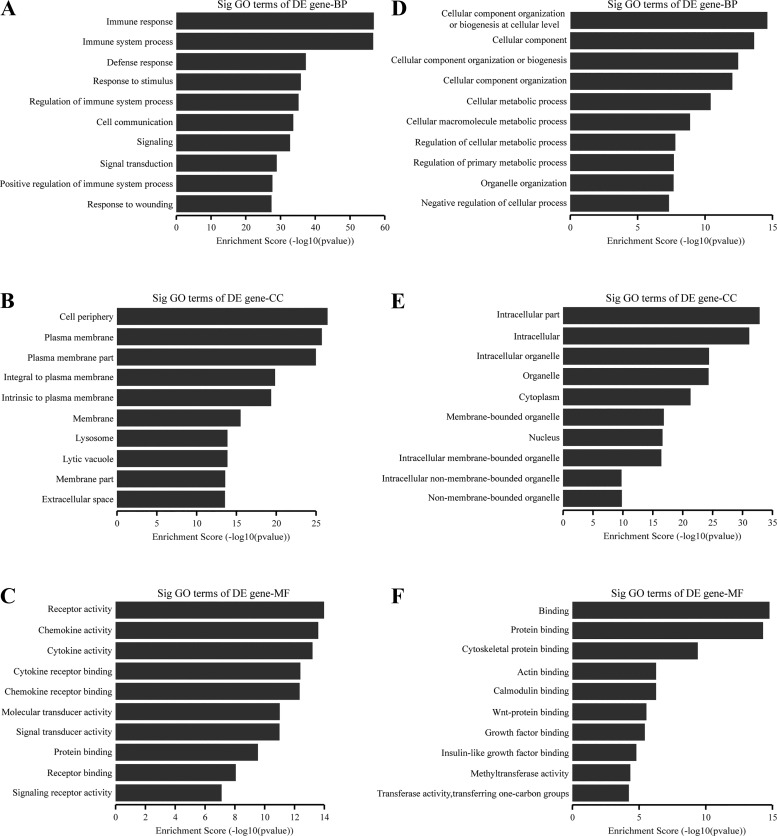
Top 10 significantly enriched GO categories for upregulated mRNAs (*A–C*) and downregulated mRNAs (*D–F*). *A, D*: biological process (BP); *B, E*: cellular component (CC); *C, F*: molecular function (MF). DE, differentially expressed.

GO analysis revealed the functions of differentially expressed mRNAs. It covers three domains: biological process, cellular component, and molecular function. We first investigated the upregulated mRNAs. In terms of biological process (Fig. 2*A*), the most significant categories were immune response, immune system process, defense response, and related immune functions. The top three categories of the cellular component (Fig. 2*B*) were cell periphery, plasma membrane, and plasma membrane part. With regard to molecular function (Fig. 2*C*), the most enriched molecules were receptors, chemokines, and cytokines. The most downregulated mRNAs were involved in cellular component organization or biogenesis at the cellular level (biological process), intracellular part (cellular component), and binding (molecular function; Fig. 2, *D*–*F*).

Significant pathways of differentially expressed genes were compared with the Kyoto Encyclopedia of Genes and Genomes (KEGG) database to further specify and identify target mRNAs. Pathway analysis showed that 65 pathways corresponded to upregulated mRNAs and 52 pathways corresponded to downregulated mRNAs. The top 10 enriched pathways for upregulated and downregulated mRNAs are shown in Supplementary Fig. S2, A and B, respectively.

#### IPA and network build.

**Fig. 3.**
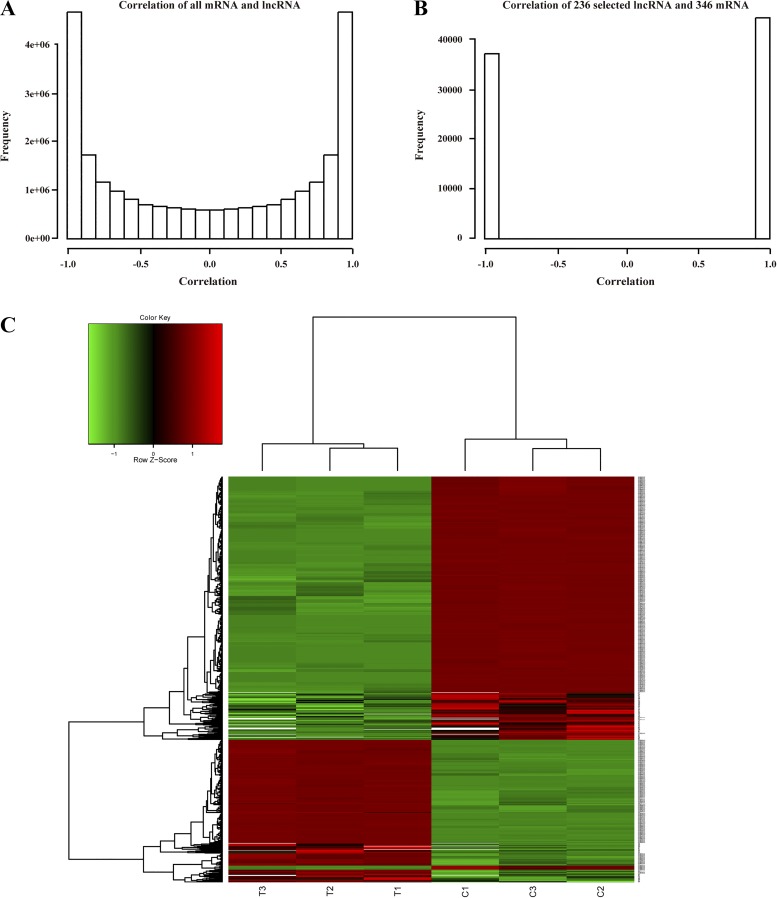
Correlation analysis of lncRNAs and mRNAs. Correlation of lncRNAs and mRNAs before (*A*) and after selection (*B*) and hierarchical clustering based on the selected 236 lncRNAs and mRNAs (*C*).

After removing duplicates and no values, we selected a total of 17,173 lncRNAs and 18,972 mRNAs for further analysis. Without any filtering, these lncRNAs and mRNAs showed small correlations (Fig. 3*A*). We then focused on differentially expressed lncRNAs and mRNA. The criteria were as follows: absolute fold change >2.5 and *P* value <0.01. After correlation analysis, we selected a total of 346 mRNAs and 236 lncRNAs that showed high correlations to one another (+1, positive correlation; −1, negative correlation; Fig. 3*B*), indicating the presence of enriched signaling between the biological genes and lncRNAs. Hierarchical clustering based on the selected 236 lncRNAs and 346 mRNAs is shown in Fig. 3*C*. Moreover, we selected an additional 142 mRNAs transcripts located near to the differentially expressed lncRNAs (within 100k bp) that may function as cis-regulated mRNAs. A total of 488 mRNAs constitute our gene set for function/pathway analysis by IPA. The results indicate that these mRNAs participated in various biological processes (Supplementary Fig. S3), such as cellular movement, cancer, cardiovascular development, and cardiovascular disease, etc. Upstream regulators such as transcription factors, cytokines, and kinases play important roles in the development and progression of arteriosclerosis. More information about these key genes from the upstream factor list, as well as the networks they regulate, is provided in Supplementary Table S3.

We continued our analysis by building networks according to these 488 selected genes. There was evidence indicating connections among these molecules in 25 networks. The highest scoring gene networks were associated with cellular growth and proliferation, cancer, and respiratory disease (Supplementary Table S4). Adhesion and migration genes such as integrin are also involved in these networks. The top six networks are shown in Supplementary Fig. S4. Among them, *network 4* was associated with cardiovascular disease, cardiovascular system development and function, and cellular development (Supplementary Fig. S4D).

#### Coexpression analysis of lncRNA and nearby protein-coding genes.

An increasing number of studies indicate that lncRNA function can be inferred from its location and will probably affect the expression of nearby protein-coding genes (2, 40). To demonstrate their interrelationships, a total of 197 lncRNAs and 180 mRNAs were chosen to perform the coexpression analysis (Supplementary Fig. S5). This figure shows that a gene may be associated with multiple neighboring lncRNAs and vice versa. Detailed information about these probes is presented in Supplementary Table S5. The top 10 upregulated and downregulated lncRNAs in this analysis are also listed in Table 3. Additionally, we found 5,279 pairs of upregulated differentially expressed lincRNAs and nearby coding genes and 5,201 pairs of downregulated lincRNAs and nearby coding genes (distance <300 kb, fold change >2, *P* < 0.05). Based on the fold change of lincRNAs, we have listed the top 10 differentially expressed upregulated and downregulated lincRNAs (Supplementary Table S6). Moreover, some lncRNAs have a similar function as an enhancer and increase the expression of nearby protein-coding genes (12). We selected 731 pairs of upregulated and 635 pairs of downregulated enhancer lncRNAs with their nearby coding genes (distance <300 kb, fold change >2, *P* < 0.05). The top 10 differentially expressed enhancer-like lncRNAs and their nearby coding genes are shown in Supplementary Table S7.

**Table 3.** Top 10 upregulated and downregulated lncRNAs associated with nearby protein-coding genes

Seqname	Gene Symbol	Regulation	*P* Value	Absolute Fold Change	RNA Length	Chromosome	Strand	Relationship	Associated Gene Name
uc001gzl.3	BC034684	up	2.59323E-06	885.5702623	460	chr1	−	exon sense-overlapping	CHI3L1, MYBPH
ENST00000443523	RP11-556E13.1	up	0.000172926	93.78290522	369	chr10	−	intergenic	MBL2
ENST00000569037	RP11-212I21.2	up	5.44075E-09	89.72674683	553	chr16	−	intronic antisense	MMP2, LPCAT2
ENST00000437416	RP11-100E13.1	up	2.31625E-08	89.05720113	403	chr1	−	bidirectional	CNIH3
uc001zvk.3	HMGN2P46	up	2.387E-05	77.29568107	942	chr15	+	natural antisense	SLC30A4, C15orf48
ENST00000510230	CTB-138E5.1	up	7.75597E-09	74.97468757	504	chr5	+	intronic antisense	TIFAB, H2AFY
ENST00000419428	RP11-356N1.2	up	2.13188E-07	67.12819909	432	chr1	+	intergenic	SLC25A34
NR_026797	LINC00520	up	4.82653E-06	62.97249688	2030	chr14	−	intergenic	KTN1
ENST00000561362	RP11-20G13.3	up	1.20159E-07	59.73290992	323	chr15	−	intergenic	LRRC28
TCONS_00001088	XLOC_000332	up	3.4218E-05	52.19734423	360	chr1	+	intergenic	SLC25A34
ENST00000453118	RP1-163G9.1	down	5.21846E-07	347.0138096	1697	chr1	−	intergenic	PRDM16
ENST00000562459	CTD-3064M3.3	down	5.75701E-06	321.3428213	1963	chr8	−	intergenic	PTP4A3
ENST00000502941	RP11-471J12.1	down	4.04957E-07	238.7432554	358	chr4	+	bidirectional	HAND2
ENST00000564832	RP11-531A24.3	down	5.11986E-06	222.4989781	3296	chr8	+	intergenic	KCNB2
NR_027180	MIR143HG	down	2.24646E-08	205.9827099	9070	chr5	+	intergenic	AFAP1L1
ENST00000563424	RP11-401P9.4	down	2.93404E-07	195.7111309	3441	chr16	+	intergenic	SNX20
NR_024617	PART1	down	3.45368E-10	173.8460326	2495	chr5	+	natural antisense	PDE4D, DEPDC1B
uc001gvp.1	C1orf81	down	7.06008E-09	163.428162	2284	chr1	+	intergenic	CACNA1S
ENST00000507525	RP13-577H12.2	down	7.78948E-08	137.4648383	233	chr4	−	natural antisense	ADAM29
uc001djh.1	BC037304	down	1.12376E-05	132.8513766	2715	chr1	+	exon sense-overlapping	PRKACB, TTLL7

#### Validation of selected lncRNAs and mRNAs by quantitative RT-PCR.

**Fig. 4.**
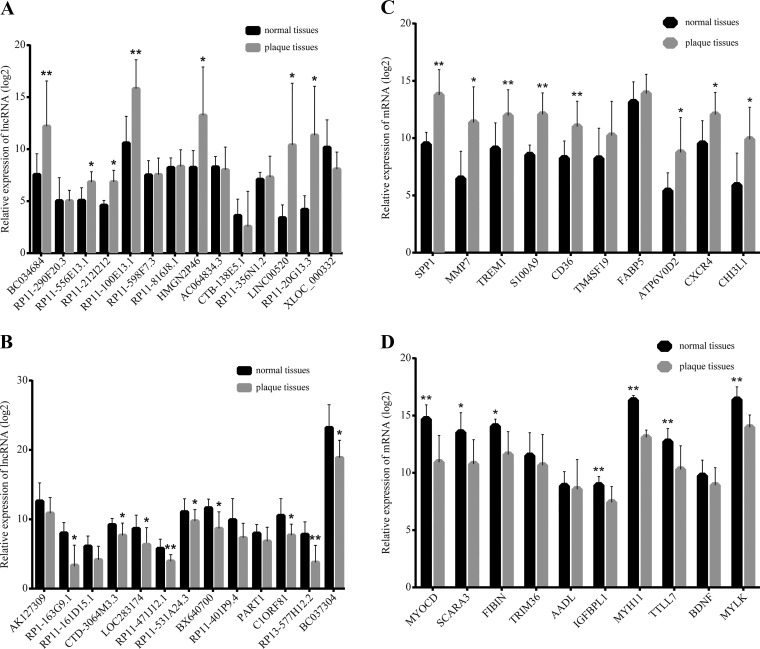
Validation of selected lncRNAs and mRNAs by quantitative RT-PCR. The relative expression levels of each selected lncRNA and mRNA were normalized and displayed in the histograms as means ± SD, *n* > 5 per group (*A*, upregulated lncRNAs; *B*, downregulated lncRNAs; *C*, upregulated mRNAs; *D*, downregulated mRNAs). All experiments were performed in triplicate.*P < 0.05, **P < 0.01 comparing plaque tissues with normal intimae.

To validate our microarray analysis findings, we performed quantitative (q)RT-PCR of a selection of 28 lncRNAs and 20 mRNAs (listed in Tables 1–3) from 10 normal intimae and 15 atherosclerotic plaques. Sixteen lncRNAs and 15 mRNAs showed high consistency between the microarray and RT-PCR results and significantly differed in their expression levels between normal intimae and atherosclerotic plaques. Among the dysregulated expression of 16 lncRNAs, 7 upregulated (Fig. 4*A*) and 9 downregulated lncRNAs (Fig. 4*B*) were identified. In addition, eight mRNAs were found to be upregulated in plaque tissues compared with normal intimae including secreted phosphoprotein 1 (SPP1), MMP7, triggering receptor expressed on myeloid cells 1 (TREM1), S100 calcium-binding protein A9 (S100A9), CD36, ATPase, H^+^ transporting, lysosomal V0 subunit D2 (ATP6V0D2), C-X-C motif chemokine receptor 4 (CXCR4), and chitinase 3-like 1 (CHI3L1) (Fig. 4*C*). Seven mRNAs were downregulated including myocardin (MYOCD), scavenger receptor class A member 3 (SCARA3), fin bud initiation factor homolog precursor (FIBIN), insulin-like growth factor binding protein-like 1 (IGFBPL1), myosin heavy chain 11 (MYH11), tubulin tyrosine ligase-like 7 (TTLL7), and myosin light chain kinase (MYLK; Fig. 4*D*).

#### Detection of human serum levels of selected proteins by ELISA.

**Fig. 5.**
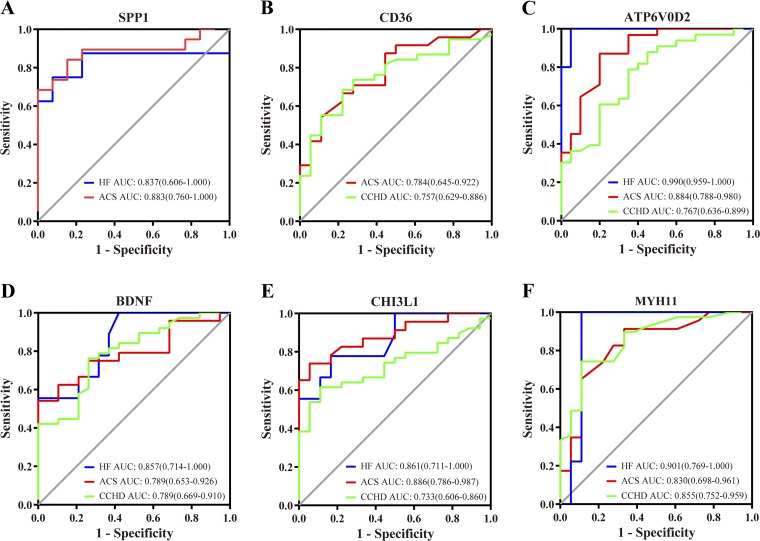
Receiver operating characteristic (ROC) curve analysis of proteins in serum samples from patients with heart failure (HF), acute coronary syndrome (ACS), chronic coronary heart disease (CCHD), and healthy controls. *A*: ROC curves for distinguishing HF patients (*n* = 75), ACS patients (*n* = 93) from healthy controls (*n* = 103) utilizing SSP1. *B*: ROC curves for distinguishing ACS patients (*n* = 93) and CCHD patients (*n* = 108) from healthy controls (*n* = 103) by CD36. ROC curves for distinguishing HF (*n* = 75), ACS (*n* = 93), and CCHD patients (*n* = 108) from healthy controls (*n* = 103), respectively, using ATP6V0D2 (*C*), BDNF (*D*), CHI3L1 (*E*), and MYH11 (*F*).

Next, we performed ELISA to investigate the corresponding protein levels of selected genes in serum samples from patients with CCHD, ACS, or HF and from healthy individuals. Table 4 shows the serum levels of these proteins and the levels of a number of biochemical measurements including TC, TG, HDL, LDL, ApoA-1, ApoB, LPa, HCY, Myo, cTnI, CK, CK-MBm, CK-MB, CRP, LDH, and AST. Furthermore, we evaluated the relationship between serum concentration of selected proteins and clinical biochemical indices of study subjects (Table 5). The specificity and sensitivity of selected differentially expressed protein measurements were assessed by ROC curves as shown in Fig. 5. The serum levels of SPP1 in patients with HF and ACS were markedly higher than those in the healthy group. Moreover, SPP1 was negatively correlated with ApoA-1 in the healthy group and positively correlated with CRP in ACS patients (Table 5). When HF patients and ACS patients were separately compared with the healthy group, the area under the curve (AUC) for SPP1 was 0.837 (*P* = 0.011) and 0.883 (*P* < 0.001), respectively. CD36 were significantly higher in patients with CCHD or ACS (*P* < 0.01). The expression level of CD36 was positively correlated with ApoB in HF patients, and its AUC was 0.784 (*P* = 0.002) when healthy individuals and ACS patients were evaluated and 0.757 (*P* = 0.002) when healthy individuals and CCHD patients were evaluated. In additional, four protein in patients with HF, ACS, or CCHD, which include ATP6VOD2, CHI3L1, MYH11, and brain-derived neurotrophic factor (BDNF), were significantly increased compared with the healthy group, respectively (all at *P* < 0.05; Table 4). Their AUC values are separately exhibited in Fig. 5, showing favorable diagnostic performance. Moreover, serum CHI3L1 levels were positively associated with HCY in the HF group and with Myo as well as CRP in ACS patients. The positive connections were found between MYH11 with LDL, ApoB, and CK in CCHD patients (all at *P* < 0.05). Additionally, BDNF was negatively correlated with CK-MB in the healthy group and with Myo in the CCHD. Several selected proteins were associated with cardiovascular disease in logistic regression models adjusted for age, sex, and known cardiovascular risk factors (Table 6). The expression of CHI3L1, CD36, and ATP6V0D2 was significantly associated with the increased risk of ACS and CCHD (all at *P* < 0.05),while the MYH11 level was slightly connected with the risk of CCHD (OR 1.002, 95% CI 1.000–1.005, *P* = 0.039). On the other hand, the ACADL level was inversely associated with the risk of HF (OR 0.763, 95% CI 0.597–0.975, *P* = 0.03; Table 6).

**Table 4.** Serum concentration of selected proteins and clinical biochemical indices of study subjects

	Healthy Group (*n* = 103)	HF Group (*n* = 75)	ACS Group (*n* = 93)	CCHD Group (*n* = 108)
TC, mmol/L	4.71 ± 0.49	3.77 ± 0.98	4.66 ± 1.48	4.34 ± 1.21
TG, mmol/L	1.05 ± 0.36	1.21 ± 0.62	1.83 ± 1.28‡	1.64 ± 0.98†
HDL, mmol/L	1.30 ± 0.19	0.99 ± 0.31‡	0.96 ± 0.26‡	1.00 ± 0.30‡
LDL, mmol/L	2.78 ± 0.39	2.35 ± 0.59	2.94 ± 1.11	2.71 ± 0.97
AopA-1, g/L	1.37 ± 0.31	1.04 ± 0.23‡	1.09 ± 0.23‡	1.13 ± 0.27‡
ApoB, g/L	0.82 ± 0.16	0.80 ± 0.25	0.90 ± 0.29	0.87 ± 0.30
LPa, g/L	0.11 (0.00,0.60)	0.26 (0.02,1.22)*	0.22 (0.01,1.22)†	0.22 (0.00,1.12)†
HCY, μmol/L	19.70 (8.60,184.00)	22.60 (9.10,36.30)*	17.15 (7.00,35.80)	19.85 (0.00,38.50)
Myo, ng/mL	26.62 (15.54,81.37)	56.30 (0.00,576.39)‡	56.36 (25.74,1000.00)‡	50.71 (17.93,819.04)‡
CTnI, ng/mL	0.00 (0.00,0.04)	0.07 (0.00,6.39)‡	3.79 (0.00,49.00)‡	0.02 (0.00,49.00)‡
CK, U/L	77 (6,912)	84 (25,1243)	168 (13,5573)‡	88 (2.88,2680)*
CK-MBm, ng/mL	0.01 (0.00,1.58)	1.61 (0.00,12.00)‡	2.18 (0.00,246.00)‡	1.04 (0.00,109.62)‡
CK-MB, U/L	11.00 (1.00,143.00)	12.50 (4.00,35.00)	20.50 (4.00,462.00)‡	14.00 (5.00,166.00)
CRP, mg/L	0.65 (0.01,257.00)	11.90 (0.09,120.70)‡	5.03 (0.20,178.70)‡	1.90 (0.08,156.10)‡
LDH, U/L	190 (80,365)	240.50 (0,790)†	265 (127,1111)‡	168 (77,1181)
AST, U/L	22 (16,105)	30 (9,242)	35.5 (10,343)†	21 (10,1104)
SPP1, ng/mL	1.50 (0.18,9.08)	19.17 (0.12,70.88)†	22.87 (0.55,120.20)‡	4.90 (0.23,226.99)
MMP7, ng/mL	5.06 (0.32,19.85)	2.06 (0.32,7.16)	5.69 (0.32,15.24)	2.06 (0.32.104.00)
TREM1, pg/mL	104.47 (63.69,324.78)	129.88 (86.42,351.10)	119.89 (55.16,920.45)	115.07 (56.34,9888.96)
S100A9, pg/mL	1086.08 (716.48,1679.37)	1071.11 (504.91,4381.86)	1045.59 (590.85,3103.03)	1086.13 (590.85,28213.91)
CD36, ng/mL	1902.45 ± 962.81	2037.25 ± 1445.03	4065.74 ± 2845.85†	3382.15 ± 1820.44 †
TM4SF19, pg/mL	56.19 (43.37,216.89)	80.50 (46.79,169.68)	89.61 (43.50,7660.00)	116.70 (42.28,375.93)
FABP5, ng/mL	1.20 ± 0.50	1.06 ± 0.33	1.23 ± 0.68	1.00 ± 0.58
ATP6V0D2, ng/mL	11.32 ± 1.08	14.45 ± 1.10*	13.44 ± 1.68†	13.00 ± 2.92*
CXCR4, pg/mL	178.26 (75.41,599.89)	164.62 (89.71,882.16)	184.72 (52.30,845.48)	205.41 (40.34,3623.1)
CHI3L1, ng/mL	32.94 ± 8.44	50.81 ± 14.35*	67.48 ± 28.53‡	52.80 ± 27.93†
MYOCD, nmol/mL	4.94 ± 0.38	6.01 ± 1.35	4.17 ± 0.57	4.55 ± 0.39
SCARA3, ng/mL	0.58 (0.41,2.15)	0.81 (0.47,1.79)	0.67 (0.001,15.75)	1.05 (0.34,5.50)
FIBIN, ng/mL	8.33 (6.43,25.59)	9.49 (6.14,15.46)	10.99 (5.64,2030.08)	10.28 (6.09,26.10)
TRIM36, ng/mL	1.78 ± 0.37	1.61 ± 0.28	1.87 ± 0.84	1.88 ± 0.36
ACADL, ng/mL	18.60 (11.09,56.06)	13.30 (10.36,20.54)	17.06 (8.52,35.10)	13.66 (6.08,142.10)
IGFBPL1, ng/mL	1.69 (0.70,2.94)	2.75 (0.92,13.32)	2.06 (0.64,11.47)	1.48 (0.70,7.38)
MYH11, pg/mL	341.57 (239.47,1786.18)	666.46 (481.04,1559.76)‡	617.55 (305.71,6767.05)‡	823.25 (288.47,8571.14)‡
TTLL7, ng/mL	0.95 (0.45,4.00)	1.24 (0.42,2.52)	1.55 (0.45,3.69)	1.41 (0.48,3.76)
BDNF, pg/mL	4308.07 ± 903.54	2939.72 ± 955.59*	3066.50 ± 1260.44†	3193.47 ± 1091.37†
MYLK, ng/mL	55.55 (4.86,289.35)	66.17 (28.79,230.54)	70.94 (18.34,402.53)	76.67 (14.25,314.84)
TC, mmol/L	4.71 ± 0.49	3.77 ± 0.98	4.66 ± 1.48	4.34 ± 1.21
TG, mmol/L	1.05 ± 0.36	1.21 ± 0.62	1.83 ± 1.28‡	1.64 ± 0.98†
HDL, mmol/L	1.30 ± 0.19	0.99 ± 0.31‡	0.96 ± 0.26‡	1.00 ± 0.30‡
LDL, mmol/L	2.78 ± 0.39	2.35 ± 0.59	2.94 ± 1.11	2.71 ± 0.97
AopA-1, g/L	1.37 ± 0.31	1.04 ± 0.23‡	1.09 ± 0.23‡	1.13 ± 0.27‡
ApoB, g/L	0.82 ± 0.16	0.80 ± 0.25	0.90 ± 0.29	0.87 ± 0.30
LPa, g/L	0.11 (0.00,0.60)	0.26 (0.02,1.22)*	0.22 (0.01,1.22)†	0.22 (0.00,1.12)†
HCY, μmol/L	19.70 (8.60,184.00)	22.60 (9.10,36.30)*	17.15 (7.00,35.80)	19.85 (0.00,38.50)
Myo, ng/mL	26.62 (15.54,81.37)	56.30 (0.00,576.39)‡	56.36 (25.74,1000.00)‡	50.71 (17.93,819.04)‡
CTnI, ng/mL	0.00 (0.00,0.04)	0.07 (0.00,6.39)‡	3.79 (0.00,49.00)‡	0.02 (0.00,49.00)‡
CK, U/L	77 (6,912)	84 (25,1243)	168 (13,5573)‡	88 (2.88,2680)*
CK-MBm, ng/mL	0.01 (0.00,1.58)	1.61 (0.00,12.00)‡	2.18 (0.00,246.00)‡	1.04 (0.00,109.62)‡
CK-MB, U/L	11.00 (1.00,143.00)	12.50 (4.00,35.00)	20.50 (4.00,462.00)‡	14.00 (5.00,166.00)
CRP, mg/L	0.65 (0.01,257.00)	11.90 (0.09,120.70)‡	5.03 (0.20,178.70)‡	1.90 (0.08,156.10)‡
LDH, U/L	190 (80,365)	240.50 (0,790)†	265 (127,1111)‡	168 (77,1181)
AST, U/L	22 (16,105)	30 (9,242)	35.5 (10,343)†	21 (10,1104)
SPP1, ng/mL	1.50 (0.18,9.08)	19.17 (0.12,70.88)†	22.87 (0.55,120.20)‡	4.90 (0.23,226.99)
MMP7, ng/mL	5.06 (0.32,19.85)	2.06 (0.32,7.16)	5.69 (0.32,15.24)	2.06 (0.32.104.00)
TREM1, pg/mL	104.47 (63.69,324.78)	129.88 (86.42,351.10)	119.89 (55.16,920.45)	115.07 (56.34,9888.96)
S100A9, pg/mL	1086.08 (716.48,1679.37)	1071.11 (504.91,4381.86)	1045.59 (590.85,3103.03)	1086.13 (590.85,28213.91)
CD36, ng/mL	1902.45 ± 962.81	2037.25 ± 1445.03	4065.74 ± 2845.85†	3382.15 ± 1820.44 †
TM4SF19, pg/mL	56.19 (43.37,216.89)	80.50 (46.79,169.68)	89.61 (43.50,7660.00)	116.70 (42.28,375.93)
FABP5, ng/mL	1.20 ± 0.50	1.06 ± 0.33	1.23 ± 0.68	1.00 ± 0.58
ATP6V0D2, ng/mL	11.32 ± 1.08	14.45 ± 1.10*	13.44 ± 1.68†	13.00 ± 2.92*
CXCR4, pg/mL	178.26 (75.41,599.89)	164.62 (89.71,882.16)	184.72 (52.30,845.48)	205.41 (40.34,3623.1)
CHI3L1, ng/mL	32.94 ± 8.44	50.81 ± 14.35*	67.48 ± 28.53‡	52.80 ± 27.93†
MYOCD, nmol/mL	4.94 ± 0.38	6.01 ± 1.35	4.17 ± 0.57	4.55 ± 0.39
SCARA3, ng/mL	0.58 (0.41,2.15)	0.81 (0.47,1.79)	0.67 (0.001,15.75)	1.05 (0.34,5.50)
FIBIN, ng/mL	8.33 (6.43,25.59)	9.49 (6.14,15.46)	10.99 (5.64,2030.08)	10.28 (6.09,26.10)
TRIM36, ng/mL	1.78 ± 0.37	1.61 ± 0.28	1.87 ± 0.84	1.88 ± 0.36
ACADL, ng/mL	18.60 (11.09,56.06)	13.30 (10.36,20.54)	17.06 (8.52,35.10)	13.66 (6.08,142.10)
IGFBPL1, ng/mL	1.69 (0.70,2.94)	2.75 (0.92,13.32)	2.06 (0.64,11.47)	1.48 (0.70,7.38)
MYH11, pg/mL	341.57 (239.47,1786.18)	666.46 (481.04,1559.76)‡	617.55 (305.71,6767.05)‡	823.25 (288.47,8571.14)‡
TTLL7, ng/mL	0.95 (0.45,4.00)	1.24 (0.42,2.52)	1.55 (0.45,3.69)	1.41 (0.48,3.76)
BDNF, pg/mL	4308.07 ± 903.54	2939.72 ± 955.59*	3066.50 ± 1260.44†	3193.47 ± 1091.37†
MYLK, ng/mL	55.55 (4.86,289.35)	66.17 (28.79,230.54)	70.94 (18.34,402.53)	76.67 (14.25,314.84)

Data are presented as means ± SD or medians (interquartile range) unless otherwise indicated. ACS, acute coronary syndrome; CCHD, chronic coronary heart disease; HF, heart failure. Compared with healthy group:

* *P* < 0.05;

† *P* < 0.01;

‡ *P* < 0.001.

**Table 5.** Correlation between serum concentration of selected proteins and clinical biochemical indices of study subjects

Selected Protein	Group	Correlated Clinical Biochemical Index	*r*	*P* Value
SPP1	Healthy	AopA-1, g/L	−0.582	0.037
SPP1	ACS	CRP, mg/L	0.637	0.003
CD36	HF	ApoB, g/L	0.734	0.024
ATP6V0D2	Healthy	AopA-1, g/L	−0.501	0.024
ATP6V0D2	HF	LDL, mmol/L	−0.975	0.005
ATP6V0D2	HF	CK-MB, U/L	−0.975	0.005
ATP6V0D2	ACS	HDL, mmol/L	−0.579	0.004
ATP6V0D2	ACS	AopA-1, g/L	−0.493	0.017
ATP6V0D2	CCHD	LDL, mmol/L	0.323	0.045
ATP6V0D2	CCHD	CK, U/L	0.365	0.022
CHI3L1	HF	HCY, μmol/L	0.795	0.010
CHI3L1	ACS	Myo, ng/mL	0.420	0.046
CHI3L1	ACS	CRP, mg/L	0.495	0.016
MYH11	CCHD	LDL, mmol/L	0.323	0.045
MYH11	CCHD	ApoB, g/L	0.321	0.046
MYH11	CCHD	CK, U/L	0.365	0.022
BDNF	Healthy	CK-MB, U/L	−0.503	0.028
BDNF	CCHD	Myo, ng/mL	−0.362	0.025
MMP7	HF	CK, U/L	0.681	0.044
MMP7	HF	CK-MB, U/L	0.773	0.015
MMP7	CCHD	TG, mmol/L	0.322	0.048
MMP7	CCHD	HDL, mmol/L	−0.325	0.047
TREM1	CCHD	HCY, μmol/L	0.431	0.007
S100A9	CCHD	TC, mmol/L	0.376	0.020
S100A9	CCHD	LDL, mmol/L	0.378	0.019
TM4SF19	HF	TG, mmol/L	0.738	0.037
TM4SF19	HF	AopA-1, g/L	0.714	0.047
TM4SF19	HF	HCY, μmol/L	−0.810	0.015
FABP5	Healthy	LDL, mmol/L	0.617	0.006
FABP5	Healthy	CK-MB, U/L	−0.569	0.014
FABP5	HF	CK-MB, U/L	−0.672	0.047
FABP5	ACS	TG, mmol/L	0.430	0.041
FABP5	CCHD	AopA-1, g/L	−0.368	0.023
CXCR4	HF	LDL, mmol/L	0.667	0.050
CXCR4	ACS	TC, mmol/L	0.522	0.011
CXCR4	ACS	TG, mmol/L	0.579	0.044
CXCR4	ACS	LDL, mmol/L	0.420	0.046
CXCR4	ACS	ApoB, g/L	0.472	0.023
CXCR4	ACS	HCY, μmol/L	0.536	0.008
CXCR4	ACS	CRP, mg/L	0.508	0.013
CXCR4	CCHD	TC, mmol/L	0.386	0.017
CXCR4	CCHD	LDL, mmol/L	0.398	0.013
CXCR4	CCHD	ApoB, g/L	0.417	0.009
MYOCD	Healthy	TG, mmol/L	0.494	0.037
MYOCD	ACS	LDL, mmol/L	0.475	0.019
MYOCD	ACS	ApoB, g/L	0.454	0.026
MYOCD	ACS	CRP, mg/L	0.527	0.008
MYOCD	CCHD	HCY, μmol/L	0.346	0.034
SCARA3	Healthy	CK, U/L	0.661	0.038
SCARA3	HF	TG, mmol/L	0.814	0.014
SCARA3	HF	AST, U/L	−0.790	0.020
SCARA3	CCHD	CTnI, ng/mL	−0.643	0.018
IGFBPL1	ACS	TG, mmol/L	0.441	0.035
IGFBPL1	CCHD	CK, U/L	0.359	0.027
IGFBPL1	CCHD	LDH, U/L	0.383	0.018
TTLL7	Healthy	HDL, mmol/L	0.717	0.020
TTLL7	HF	AopA-1, g/L	0.881	0.004
TTLL7	HF	HCY, μmol/L	−0.833	0.010
TTLL7	CCHD	CK-MB, U/L	−0.554	0.050
TTLL7	CCHD	AST, U/L	−0.626	0.022
MYLK	Healthy	HCY, μmol/L	0.511	0.030
MYLK	HF	LDL, mmol/L	0.800	0.010
MYLK	HF	ApoB, g/L	0.683	0.042
MYLK	ACS	TC, mmol/L	0.488	0.018
MYLK	ACS	TG, mmol/L	0.468	0.024
MYLK	ACS	ApoB, g/L	0.501	0.015
MYLK	ACS	HCY, μmol/L	0.462	0.026
MYLK	ACS	CRP, mg/L	0.530	0.009

**Table 6.** Association of risk factors with HF, ACS, CCHD, and healthy controls

Risk factor	Disease	Odds Ratio (99% CI)	*P* Value
BDNF	HF	0.998 (0.997–1.000)	0.013
BDNF	ACS	0.999 (0.998–1.000)	0.004
BDNF	CCHD	0.999 (0.998–1.000)	0.002
MYH11	CCHD	1.002 (1.000–1.005)	0.039
CD36	ACS	1.001 (1.000–1.001)	0.016
CD36	CCHD	1.001 (1.000–1.001)	0.006
CHI3L1	HF	1.144 (1.033–1.267)	0.01
CHI3L1	ACS	1.122 (1.040–1.210)	0.003
CHI3L1	CCHD	1.068 (1.016–1.123)	0.01
ATP6V0OD	ACS	5.210 (2.031–13.361)	0.001
ATP6V0OD	CCHD	2.346 (1.292–4.259)	0.005
ACADL	HF	0.763 (0.597–0.975)	0.03

## DISCUSSION

Atherosclerosis, the underlying cause of cardiovascular disease, represents the leading cause of morbidity and mortality throughout industrialized societies (3, 17). Due to the complexity of its pathogenesis, effective diagnosis and therapeutic strategies have not yet been applied in routine clinical practice. We focused on plaques obtained from patients and investigated differentially expressed RNAs by microarray analysis and verified the expression levels of those RNAs and proteins that may play important roles in atherosclerosis and other acute cardiovascular events. In our study, we investigated differences in lncRNA and mRNA profiles between advanced atherosclerotic plaques and the normal arterial intima. Given that lncRNAs can play regulatory roles through interaction with target genes, we also systematically investigated differentially expressed mRNAs. Some of these genes have been verified as pivotal to the occurrence and development of atherosclerosis and other cardiovascular diseases, but most of them and their associated lncRNAs have not been well studied, and their potential functions remain unclear. Therefore, our study provides novel information about these specific lncRNAs and target genes, which may contribute to the pathogenesis of atherosclerosis and identifies potential therapeutic targets.

Numerous dysregulated lncRNAs and mRNAs in plaques tissues were detected by performing microarray profiling. We systematically investigated these differentially expressed mRNAs in their function, pathway, and network. GO functional-enrichment analysis of genes showed that several differentially expressed mRNAs were related with cytokine and chemokine activity, which participates in all phases of atherosclerotic lesion development. Additionally, other molecules involved in vascular trauma including NF-κB, alpha-catenin, vascular endothelial growth factor, and extracellular regulated protein kinases (ERK) were also activated. From function analysis, we speculate that the selected genes may be related to cardiovascular development and blood cell movement.

The pathway analysis shed light on which pathways were enriched in genes contributing to the development of advanced atherosclerotic plaques. They include the MAPK signaling pathway, Toll-like receptor signaling pathway, and NF-κ signaling pathway, which have previously been reported to be associated with atherosclerosis pathogenesis (25, 28, 32, 37). Moreover, we undertook a network analysis to predict pathways that may regulate a panel of selected genes and observed high-scoring gene networks related to cellular growth and proliferation, cancer, respiratory disease, and others. Many of these networks are associated with cardiovascular anomalies such as lipid metabolism, cardiovascular system development, and function. The molecules in these networks may play important roles in regulating the selected genes. Many of them are consistent with regulators in the upstream regulator analysis such as ERK, P38, and SPP1. The network analysis provides a direction for further function and mechanism studies and indicates potential regulatory factors and downstream genes that may play a key role in atherosclerosis.

In our analysis, a number of lncRNAs and mRNAs were found to be related to atherosclerosis. We found that SPP1, which displayed the largest fold change in the microarray analysis, was differentially expressed in the serum of patients with HF or ACS when compared with healthy controls. Studies have shown that SPP1 is an integrin-binding ligand and N-linked glycoprotein participating in atherosclerotic inflammation (35). Our results support a role for SPP1 in atherosclerosis. Additionally, we found that CD36, a scavenger receptor, was at higher levels in the serum of patients with ACS or CCHD than in normal subjects. It is widely accepted that CD36 mediates foam cell formation and promotes inflammatory response and oxidative stress (29, 39). Our results are consistent with a role for CD36 in promoting atherosclerosis. ATP6V0D2, which is required for osteoclast differentiation as well as the bone-resorptive function of mature osteoclasts (5, 20), was found to be expressed at higher levels in the serum of patients with HF, ACS, or CCHD than in the healthy group. CHI3L1, which has been widely investigated in cell proliferation, differentiation, apoptosis, angiogenesis, inflammation, and extracellular tissue remodeling (18), also had higher levels in patients with HF, ACS, or CCHD. It is also worth noting that serum levels of BDNF, which has gained strong interest for its relevance to the incidence of psychiatric syndromes and neurocognitive functioning (38), were lower in patients with CCHD, ACS, or HF than in healthy subjects. Furthermore, we found that BDNF protein levels were significantly correlated with the levels of CK-MBm and Myo, which have been widely used in the diagnosis of cardiovascular diseases. These ELISA results demonstrated that some of our selected proteins were differentially expressed not only in plaque tissues and normal intimae, but also in the serum of patients with different cardiovascular diseases and may, therefore, serve as biomarkers for the diagnosis and prognosis monitoring of diseases. In summary, our results support the conclusion that these proteins (i.e., SPP1, ATP6V0D2, CHI3L1, and BDNF) likely play important roles in the development of atherosclerosis-related diseases and further indicate that they are potential diagnostic and therapeutic biomarkers.

Our study has several limitations. First, the atherosclerotic plaques used in the microarray analysis were of grade VI, while the plaques used in the qRT-PCR validation analysis varied from grade IV to VI. Due to the complexity of plaque composition and tissue variability, the fold changes of the selected lncRNAs and mRNAs in the qRT-PCR analysis were not as large as those seen in the microarray experiment. It is likely that the expression levels of these genes vary depending on the grade of atherosclerotic plaque. Second, probably because of the insufficient sample size in our pilot study, some lncRNAs and mRNAs, which expressed significant differentially between the health group and the disease group by the chip analysis, did not show such significant differences in verification of qPCR and ELISA. So even though we expanded the sample size for qPCR validation and ELISA testing, the six tissue samples we used for pilot studies was slightly small. Third, the comparison of plaque/control sample has some limitation, as the samples come from whole tissue and do not provide information on which cell type has the highest expression; this may limit the application of the present study.

### Conclusions

In conclusion, our study provides a distinct expression profile of lncRNAs and mRNAs in advanced atherosclerotic plaques compared with normal arterial intimae and identifies the pathways involved. Furthermore, our study indicates that SPP1, CD36, ATP6V0D2, CHI3L1, MYH11, and BDNF, which showed favorable diagnostic performance and high correlations with several clinical biochemical parameters, may potentially serve as biomarkers to diagnose and monitor the prognosis of atherosclerosis.

## GRANTS

This work was supported by the National Natural Sciences Foundation of China (Grants 81871701, 81572051, and 81772244), the Natural Science Fund of Guangdong (Grants 2017A030313535, 2017A030313532, and 2018A030313533), the Science and Technology Program of Guangzhou (Grants 201607010267, 201604020015, 201707010034, 201704020213 and 201707010156), Foundation of Qingyuan Science and Technology Bureau Project 2018A009, and Guangdong Province Medical Science and Technology Research Fund Project A2015009.

## DISCLOSURES

No conflicts of interest, financial or otherwise, are declared by the authors.

## AUTHOR CONTRIBUTIONS

All authors meet the three authorship criteria of the International Committee of Medical Journal Editors. L. Zheng contributed to design, acquisition of data, analysis, and interpretation of results along with drafting and revising the article. Y.-W. Hu contributed to the design, interpretation of the data and revising for critically important intellectual content. H.-L. Bai contributed to analysis and interpretation and revising the article. J.-J. Zhao contributed to analysis and interpretation and drafting the article. The others contributed to conception and design, interpretation of the results, and critically reviewing for intellectual content. All authors knew in advance of their contribution and gave approval of the final version to be submitted.

H.-L.B., Z.-F.L., X.-H.L., S.-G.W., and Y.-J.X. performed experiments; H.-L.B., H.X., C.-M.K., J.-B.L., and Q. Wu prepared figures; H.-L.B. edited and revised manuscript; J.-J.Z. drafted manuscript; X.M., X.-H.L., S.-G.W., and L.X. analyzed data; C.-M.K., J.-B.L., Q. Wang, L.Z., and Y.-W.H. conceived and designed research; S.Y., Q. Wang, and Y.-W.H. interpreted results of experiments; L.Z. and Y.-W.H. approved final version of manuscript.
